# A low-frequency chip-scale optomechanical oscillator with 58 kHz mechanical stiffening and more than 100^th^-order stable harmonics

**DOI:** 10.1038/s41598-017-04882-4

**Published:** 2017-06-29

**Authors:** Yongjun Huang, Jaime Gonzalo Flor Flores, Ziqiang Cai, Mingbin Yu, Dim-Lee Kwong, Guangjun Wen, Layne Churchill, Chee Wei Wong

**Affiliations:** 1 School of Communication and Information Engineering, University of Electronic Science and Technology of China, Chengdu, 611731 China; 2 Fang Lu Mesoscopic Optics and Quantum Electronics Laboratory, University of California, Los Angeles, CA 90095 USA; 3 Institute of Microelectronics, A*STAR, Singapore, 117865 Singapore; 4 Georgia Tech Research Institute, Atlanta, GA 30318 USA

## Abstract

For the sensitive high-resolution force- and field-sensing applications, the large-mass microelectromechanical system (MEMS) and optomechanical cavity have been proposed to realize the sub-aN/Hz^1/2^ resolution levels. In view of the optomechanical cavity-based force- and field-sensors, the optomechanical coupling is the key parameter for achieving high sensitivity and resolution. Here we demonstrate a chip-scale optomechanical cavity with large mass which operates at ≈77.7 kHz fundamental mode and intrinsically exhibiting large optomechanical coupling of 44 GHz/nm or more, for both optical resonance modes. The mechanical stiffening range of ≈58 kHz and a more than 100^th^-order harmonics are obtained, with which the free-running frequency instability is lower than 10^−6^ at 100 ms integration time. Such results can be applied to further improve the sensing performance of the optomechanical inspired chip-scale sensors.

## Introduction

Recent years, radiation-pressure driven cavity optomechanics^1–3^ have been considered as the new research frontiers of fundamental physics and emerging applications, for examples, the laser cooling^4–6^, phonon lasers^7^, induced-transparency^8, 9^, chip-scale stable RF sources^10, 11^, and the quantum transductions of microwave, spin, and optical qubits^12, 13^. Specifically, the highly coupled optical and mechanical degrees-of-freedom^14, 15^ allow detection of nanomechanical motion^16–18^ for, such as, force sensing^19^, radio wave detection^20^, AC/DC acceleration^21–23^, as well as the field detection^24, 25^. Based on the classical thermal limit of the nanomechanical motion detection, in terms of the displacement noise^21, 26^:

1 $${S}_{xx}({\rm{\Omega }})=\frac{{S}_{FF}({\rm{\Omega }})}{{m}_{eff}^{2}}\chi ({\rm{\Omega }})=\frac{4{k}_{B}T{{\rm{\Omega }}}_{m}}{{m}_{eff}{Q}_{m}}\frac{1}{{({{\rm{\Omega }}}^{2}-{{\rm{\Omega }}}_{m}^{2})}^{2}+{({{\rm{\Omega }}{\rm{\Omega }}}_{m}/{Q}_{m})}^{2}},$$

one has to increase the test mass of the chip-scale sensor as large as possible. Here *S*_*xx*_(Ω) and *S*_*FF*_(Ω) are the mechanical displacement spectral density and force spectral density, *m*_*eff*_ the test-mass, *χ*(Ω) the susceptibility, *k*_*B*_ the Boltzmann constant, *T* the temperature, *Q*_*m*_ the mechanical quality factor, and Ω_*m*_ the mechanical resonance angular frequency. As a result, according to the relation of Ω_*m*_^2^ = *k*/*m*_*eff*_, the fundamental mechanical oscillation modes should be at kHz or MHz levels, such as the recently reported chip-scale optomechanical accelerometer^21–23^ and magnetometer^24, 25^.

Among most of the chip-scale optomechanical cavity platforms, e.g., micro-toroid^27^, micro-disk^28^, as well as photonic crystal (PhC) zipper cavity^29^ and slot PhC cavity^30^, the PhC based optomechanical systems possess theoretically and experimentally the highest optomechanical transduction rate^31–33^. Unfortunately, for the kind of PhC inspired optomechanical cavity, one big challenge for the chip-scale large-mass sensor^21, 23^ is to keep the movable test mass on the same plane of the fixed part when released (“air-bridged”) experimentally, to obtain as large optomechanical transduction coefficient (also called as optomechanical coupling rate and defined as *g*_om_ = *dω*/*dx*) as possible. Most of the fabrication processes were not able to achieve this reliably. By using standard CMOS-compatible fabrication processing on the silicon-on-insulator platform and the high-yield chip release procedure with vapor-phase HF etcher (see Methods), here we report several improved optical and mechanical performances for the low-frequency optomechanical cavity towards the applications of force- and field-sensing. Specifically, the proposed low-frequency optomechanical oscillator (OMO) has a test mass of ≈5.6 ng which operates at ≈77.7 kHz fundamental mode and intrinsically exhibits large optomechanical coupling rate of 44 GHz/nm or more, for both two optical resonance modes. The mechanical stiffening of ≈58 kHz and more than 100^th^-order harmonics are obtained, with which the free-running frequency instability for the fundamental mode is lower than 10^−6^ at 100 ms integration time.

## Results

### Chip characteristics

**Figure 1:**
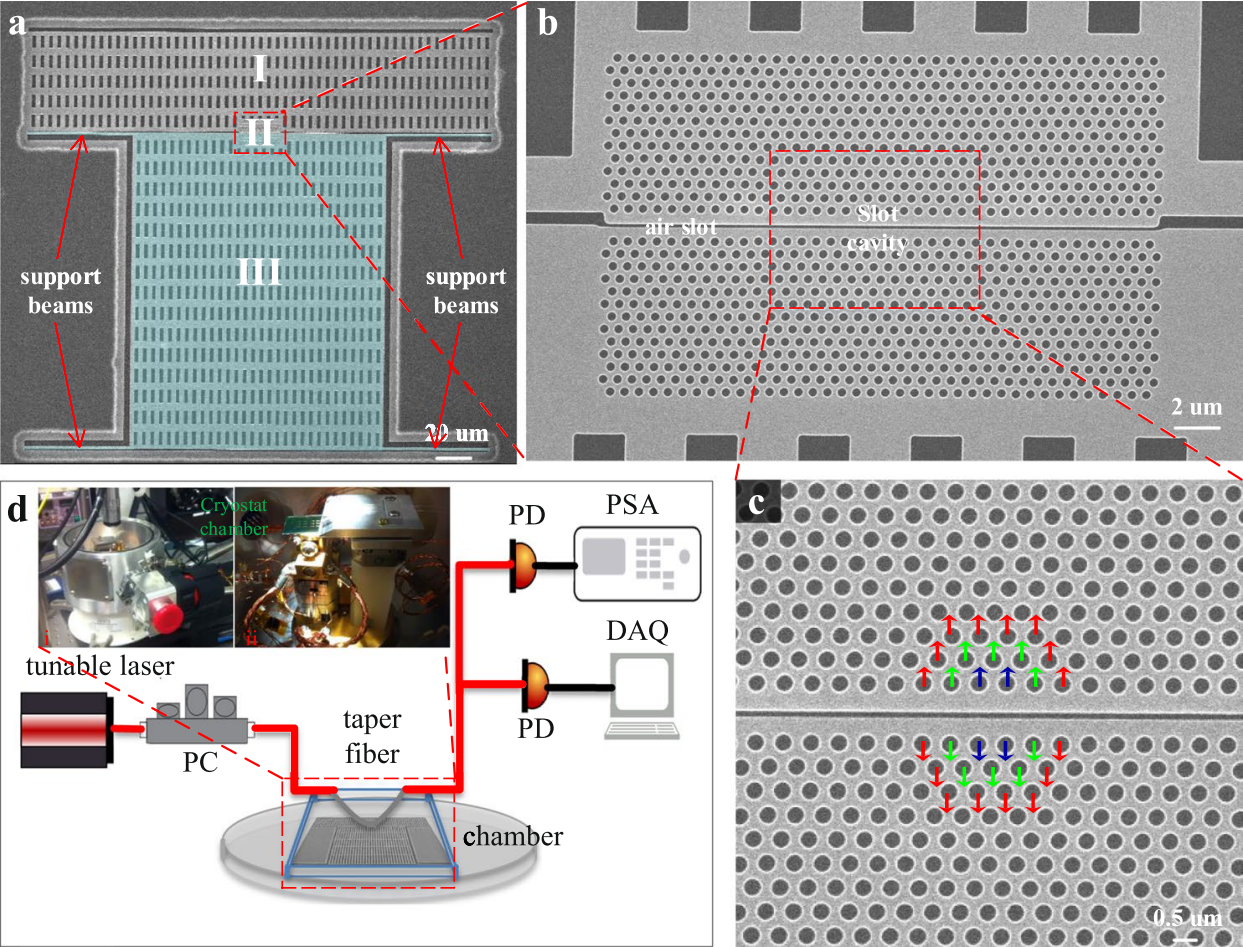
Optomechanical cavity oscillator with large moveable mass. (**a**) Scanning electron micrograph (SEM) of nanofabricated large mass silicon optomechanical oscillator. Region I: stationary part, Region II: slot cavity part, and Region III: test-mass part. Scale bar: 20 μm. (**b**) Zoom-in SEM of the photonic crystal optomechanical device. Scale bar: 2 μm. (**c**) Zoom-in SEM of the photonic crystal slot cavity with hole perturbation shifts denoted in color. Red: 5 nm, green: 10 nm, blue: 15 nm. Scale bar: 500 nm. (**d**) Measurement setup. PD: photo detector, PSA: Power Spectrum Analyzer, PC: polarization controller, and DAQ: Data Acquisition. Inset is the image of the vacuum chamber and 5-axis Attocube positioner.

Figure 1a illustrates the low-frequency optomechanical cavity nanofabricated in a silicon-on-insulator substrate with a 250-nm thick silicon layer by CMOS-compatible processes (see Methods). A slot-type PhC cavity^30, 31, 34^ is designed and located at the center region of the large test mass device (see Fig. 1b). The slot cavity features a slot width *s* of ≈100 nm, 470 nm photonic crystal lattice constants (*a*_*pc*_), and 150 nm hole radii, with 5 nm (red), 10 nm (green) and 15 nm (blue) lattice perturbations, as shown in Fig. 1c, to form the cavity mode on the line-defect PhC waveguide with width 1.2 × $\sqrt{3}$ *a*_*pc*_. The large (≈120 μm × 150 μm) 5.6 ng test-mass for force- and field-sensing has four (1 μm × 50 μm) compliant support beams, featuring a ≈77.7 kHz fundamental resonance mode and a 1.33 N/m combined stiffness. It is attached to the lower side of the slot cavity shown as in Fig. 1a. The upper side of the slot cavity is anchored to the silicon substrate and has the same *x*-length as the test-mass to reduce asymmetric residual stress *z*-bow between the two parts, to preserve the localized optical resonance mode. The same sized rectangular holes outside the PhC part are added on both the test-mass (lower side) and the fixed mass (upper side) to save the release time and improve the yield when preform the HF vaper phase release process (see Methods).

Figure 1d shows the measurement setup in which the optomechanical cavity is placed inside the vacuum chamber and probed by a dimpled tapered fiber^30, 35^. The fiber coupling is adjusted and optimized by controlling both the tapered fiber and low-frequency OMO chipset mounted on a 5-axis Attocube positioner within a customized Janis ST-500 vacuum chamber. Santec TSL-510 tunable laser is used as the driving source and the polarization state is determined by the polarization controller (PC). The optical and mechanical modes are read out by the slow and fast photodetectors, and the data are recorded by the DAQ and LabVIEW program (see Methods). For vacuum measurements, an Edwards T-Station 75 turbopump evacuates the chamber to high vacuum. The pumping station consists of an E2M1.5 backing pump and a XDD1 turbo pump, allowing the chamber to reach 10^−7^ mbar vacuum.

### Demonstration of large mechanical stiffening range

**Figure 2:**
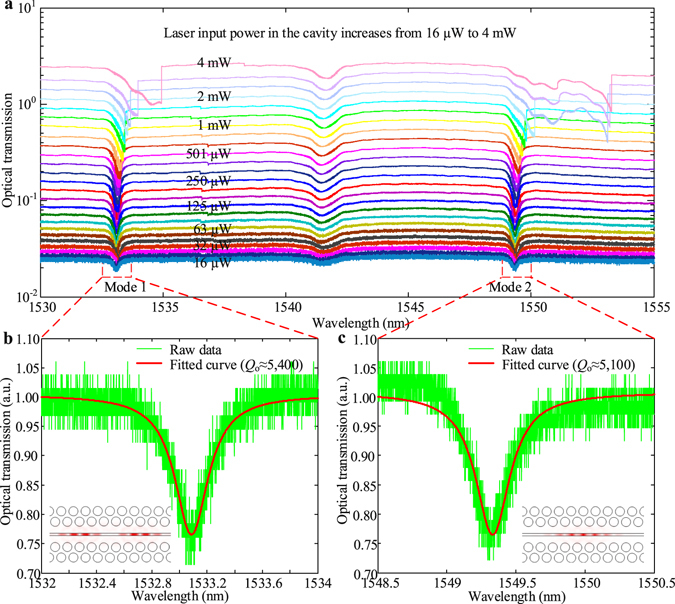
Optical transmission spectra of the optomechanical cavity. **(a**) Optical transmission spectra under different input powers from 16 μW to 4 mW. (**b** and **c**) Magnified plots for the two modes with the fitted loaded optical *Q*.

Figure 2a firstly shows the measured optical transmission spectra under different drop-in intracavity powers at atmosphere and room temperature. It is found that there are two optical modes located at ≈1533.1 nm and ≈1549.3 nm respectively. Under low-drive power (16 μW), the loaded cavity quality factor *Q*_*o*_ for both modes are measured at ≈5,400 and 5,200, as shown in Fig. 2b,c (intrinsic cavity *Q*_*in*_ at ≈7,300, and 7,100). With increasing drive powers to 4 mW, thermal nonlinearity^36^ broadens the cavity resonances into asymmetric lineshapes for both modes. The electric field distributions (|*E*_y_|^2^) of the two modes are inserted in the Fig. 2b,c, respectively, which show most of the electric energy are located at the air slot region and therefore can couple to the mechanical motion properly.

**Figure 3:**
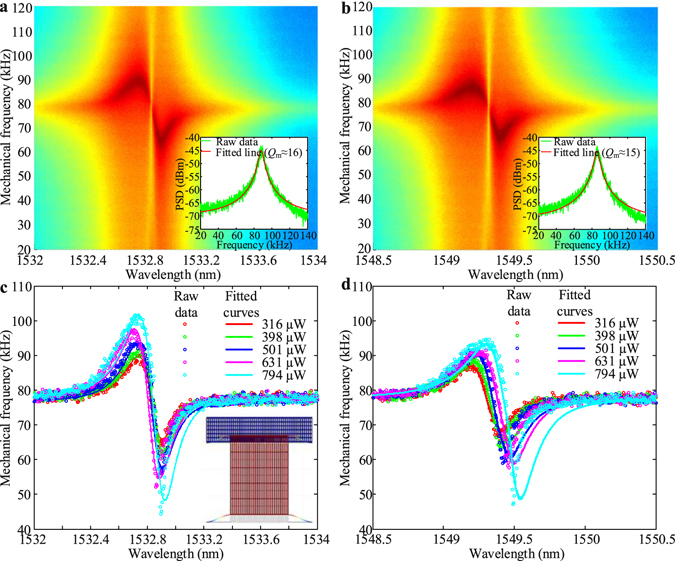
Mechanical frequency stiffening versus different optical detunings at various input powers. (**a**) and (**b**) The two-dimensional mapping plots for the mechanical frequency stiffening versus different optical detunings for mode 1 and mode 2, respectively. The drive power here is 316 μW. The insets are the power spectrum density (PSD) at blue detuning side (≈1532.65 nm and ≈1549.19 nm for the two optical modes). (**c** and **d**) The peak mechanical frequency shifts for different input powers. The solid lines are the fitted data for determining the optomechanical coupling (*g*_om_) with the optomechanical stiffening relation. The *g*_om_ for both modes are obtained as ≈43.9 GHz/nm and 49.8 GHz/nm, respectively.

Figure 3 shows the measured changing properties of the RF spectra with a swept pump wavelength at different optical drive powers for the two optical modes. It is shown that, the mechanical resonance frequency changing properties versus the swept pump wavelength indicate the optomechanical stiffening, described for example in ref. 31. In the slot-type optomechanical cavity, with the deeply sub-wavelength confinement, the optomechanical stiffening and optical-mechanical resonance spectra of the cavity are strongly dependent on the drive power level and the optomechanical coupling rate. Consequently, the optomechanical stiffening increases with a resulting modified mechanical frequency resonance Ω^′^_*m*_ which can be described by:

2 $${{\rm{\Omega }}^{\prime} }_{m}=\sqrt{{{\rm{\Omega }}}_{m}^{2}+(\frac{2{|a|}^{2}{g}_{om}^{2}}{{{\rm{\Delta }}}^{2}{\omega }_{c}{m}_{eff}}){{\rm{\Delta }}}_{o}}=\sqrt{{{\rm{\Omega }}}_{m}^{2}+(\frac{2{|a|}^{2}{g}_{om}^{2}}{({({\omega }_{l}-{\omega }_{c})}^{2}+{({\rm{\Gamma }}/2)}^{2}){\omega }_{c}{m}_{eff}})({\omega }_{l}-{\omega }_{c})},$$

where Ω′_*m*_ (Ω_*m*_) is the shifted (unperturbed) resonance frequency, |*a*|^2^ is the averaged intracavity photon energy, Γ = 1/*τ* the optical cavity decay rate, *ω*_*c*_ the optical resonance frequency, *ω*_*c*_ the frequency of the drive laser, Δ_*o*_ = *ω*_*l*_ − *ω*_c_ and Δ^2^ = ${{\rm{\Delta }}}_{o}^{2}+{({\rm{\Gamma }}/2)}^{2}$.

As shown in Fig. 3a,b, the optical resonances are 1532.82 nm and 1549.29 at 316 μW drive power with an intracavity photon number of ≈13,380 and ≈9,940 at zero optical detuning. With increased drive powers from 316 μW to 794 μW, the optomechanical stiffening increases due to the stronger optical gradient force and, as a result, the mechanical stiffening range reach to maximum 58 kHz for the first mode as shown in Fig. 3c,d. The modeled mechanical frequencies for different detunings [equation (2)] under different drive powers (corresponding to the powers in measurements) are also superimposed on the measurement (soiled lines shown in Fig. 3c,d). From the measurement-model correspondence, we obtain the optomechanical coupling rates *g*_*om*_ */*2*π* ≈ 43.9 GHz/nm and 49.8 GHz/nm, with the vacuum optomechanical coupling rate *g* ^*^ */*2*π* = 193 kHz and 219 kHz, for the two modes. We note that the measured *g*_*om*_ */2π* here is still smaller than theoretically prediction^31^ due to elevation asymmetries of the released masses, lowering the optomechanical transduction from designed values.

We note that the two optical modes in Fig. 3 have slight blue-shifts compared to the previous measured results shown in Fig. 2. This is because such two measurements are carried out in different runs, with variants on the fiber coupling. Furthermore, we note that the first mode with smaller optomechanical coupling rate has larger mechanical stiffening range while the second mode with larger optomechanical coupling rate has smaller mechanical stiffening range. This is due to fact that the intracavity photon number of first mode is larger than the second mode as demonstrated previously. Moreover, with blue optical detunings (≈1532.65 nm and 1549.19 nm for the two optical modes), the cold fundamental mechanical mode (the mechanical displacement can be found in the inset of Fig. 3c) at atmosphere and room temperature is located at ≈77.7 kHz with mechanical quality factor *Q*_*m*_ of ≈16 before driven into oscillation (see insets of Fig. 3a,b).

The small mechanical quality factor is mainly from gas damping^21^. When reducing the pressure to vacuum, the mechanical quality factor increases strongly as confirmed in refs 21 and 23, and to be ≈1380 for our device. Furthermore, with increases in the intrinsic mechanical quality factor, the power threshold to achieve oscillation state can be reduced significantly, described by the relation^27^:

3 $${{P}}_{{thresh}}\propto \frac{1}{{Q}_{m}}{(\frac{1}{{Q}_{o}})}^{3}.$$

Next, we will demonstrate the low-frequency oscillation state and the abundant harmonics in vacuum.

### Demonstration of more than 100^th^-order stable harmonics

**Figure 4:**
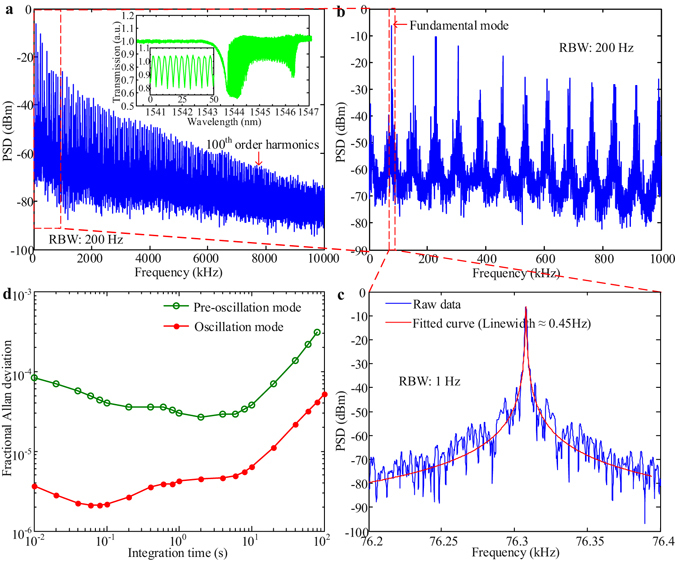
Mechanical oscillation characteristics of the low-frequency OMO at vacuum state. **(a**) More than 100^th^ order harmonics of mechanical oscillations are obtained for the low-frequency OMO. The resolution bandwidth (RBW) is 200 Hz and the video bandwidth (VBW) is RBW/3. The inset is the corresponding optical transmission indicating the self-induced regenerative oscillations with the signature spectral fluctuations. One zoom-in plot for the optical wavelength from 1545.1 nm to 1545.15 nm is embedded in the inset figure. (**b**) Zoom-in plot for the first 13 harmonics. The RBW is 200 Hz and the VBW is RBW/3. (**c**) The fundamental mode of the low-frequency OMO with fitted curve, which shows the obtained oscillation linewidth of ≈0.45 Hz. Both the RBW and VBW are 1 Hz. (**d**) The measured Allan deviation performances of the low-frequency OMO for both pre-oscillation and oscillation mode.

Driven into oscillation mode in vacuum (the drive power currently is ≈600 μW, much larger than the power threshold of ≈200 μW), we firstly observed the self-induced regenerative oscillation with the signature spectral fluctuations as shown in the inset of Fig. 4a. More than 100^th^ order harmonics are obtained as shown in Fig. 4a, due to the nonlinear optomechanical transduction from the asymmetric optical lineshape. Such abundant harmonics indicate the low-frequency OMO device can potentially serve as a RF frequency multiplier. Figure 4b shows the zoom-in first 13 harmonics in the frequency range from DC to 1 MHz, which indicates some sidebands due to the mixing with low-frequency noises and the out-of-plane modes^23^. The Lorentzian fitted linewidth of the fundamental mode is ≈0.45 Hz as shown in Fig. 4c and this measured linewidth is mostly limited by the resolution-bandwidth of the spectrum analyzer used in the experiments. We note that, when in the self-induced regenerative oscillation regime in vacuum, the optomechanical cavity oscillator has a ≈75 dB signal-to-noise compared to ≈25 dB in the resonant (pre-oscillation) mode in atmosphere. This will aid the minimum detectable frequency shift and contribute to high sensing resolution applications.

To achieve the high resolution for force- and field-detection, short and longer-term frequency instability is one key parameter which should be determined carefully. For the low-frequency OMO, here we measure the Allan deviation by using frequency counter (Hewlett-Packard 5351 A) to characterize the frequency fluctuation (see Methods) for the pre-oscillation and oscillation modes in vacuum as shown in Fig. 4d. It indicates the low-frequency OMO in oscillation mode has a fractional frequency instability δΩ_0_/Ω_c_ (Ω_c_ is the carrier frequency ≈76.31 kHz) at 4.1 × 10^−5^ and 2.2 × 10^−6^ at 100 ms integration time, for the pre-oscillation and oscillation states respectively. In oscillation state, this is equivalent to a thermal noise limited minimal detectable frequency shift δΩ_m_/2π of 167 mHz in 100 ms integration time.

## Discussion

In summary, we have demonstrated a low-frequency optomechanical oscillator which shows several improved optical and mechanical performances. By using standard CMOS-compatible fabrication process on the silicon platform and the high-yield chip release procedure with vapor phase HF etcher, the low-frequency OMO with a test mass of 5.6 ng which operates at ≈77.7 kHz in the fundamental mode and exhibits large optomechanical coupling rate of 44 GHz/nm or more, for both optical resonance modes. The mechanical stiffening of ≈58 kHz and more than 100^th^ order harmonics are obtained. The free-running frequency instability for the fundamental mode is characterized to be lower than 10^−6^ at 100 ms integration time.

The mesoscopic room-temperature implementation with RF optomechanical transduction and readout provides a new platform towards low-noise precision metrology. For examples, with increased optomechanical coupling and the intracavity photon energy, the significant increase for the frequency tuning slope |dΩ/dλ|^37^ is achieved, resulting in the improvement of the sensitivity. Moreover, with the stable mechanical oscillation reference, the sensing resolution will also be improved based on the relationships of *δ*Ω_*th*_ = [(*k*_*B*_ *T*/*E*_*C*_)(Ω_*m*_Δ*f/Q*_*m*_ *)*]^1/2^ and *R* = *S* × *δ*Ω_*th*_/2π. Here *δ*Ω_*th*_ stands for the minimum detectable mechanical frequency shift and it relates to the energy *E*_C_ stored in the cavity and the intrinsic mechanical quality factor *Q*_*m*_ of the oscillator, and Δ*f* is the measurement bandwidth.

The discussed large-mass optomechanical oscillator has been fabricated by standard CMOS-compatible process on the silicon platform and it has shown such good performances. For the potential sensing applications, our next step is to integrate the on-chip silicon waveguide to couple in the laser drive source and couple out the modulated optical powers, like the works have done previously^11, 38^.

## Methods

### Device nanofabrication

The low-frequency OMO is fabricated in the CMOS foundry (Institute of Microelectronics, Singapore). The CMOS-compatible process consists of multi-level mask alignments on an 8′′ silicon wafer with 250-nm device thickness on top of a 3 μm buried oxide cladding at the foundry (more details can be found in ref. 11). The resulting cavities are shown in Fig. 1b,c with ≈100 nm slot widths. Next all the PhC cavity and lattice patterns are aligned to the slot arrays across the 8′′ wafer and optimally etched into the device layer, to create the optomechanical cavity (unreleased).

### Device release

The PhC cavity is next carefully released by etching the bottom oxide with AMMT HF vapor phase etcher and tightly controlling process at UCLA Nanoelectronics Research Facility (Nanolab). To save the etching time and the success rate (prevent the stiction), the low-frequency OMO chipset is firstly soaked by wet HF (6:1 buffered-oxide etchant) for 10 minutes and then transferred to the HF vaper phase etcher (relative temperature is set as 12 °C) for further 100 minutes.

### Experiment setup

The low-frequency OMO chipset is mounted on a 5-axis Attocube positioner within a customized Janis ST-500 vacuum chamber (see inset of Fig. 1d). A dimpled tapered fiber with more than 90% transmission is passed through a fiber feedthrough and coupled to the device, by anchoring the fiber dimple directly on the chip surface and on the lower side (see Fig. 1a) of the optomechanical slot cavity. The optical drive is provided by a tunable laser diode (Santec TSL-510) and a fiber polarization controller is used to select the transverse-electric (TE) state-of-polarization to drive the optomechanical oscillator. While simultaneous readouts of the RF and optical spectra are done with external fast photodetector (New Focus 125 MHz detector) and slow detector (Thorlabs 125 kHz InGaAs detector). Finally, the data are recorded by the NI DAQ and LabVIEW program.

### Numerical modeling

The optical resonant modes and electromagnetic fields of the photonic crystal cavity are obtained from finite-difference time-domain simulations through a freely available software package (MEEP)^39^. Mechanical displacement fields and modes are determined with COMSOL Multiphysics. Coupled mode theory on the optical fields and the harmonic oscillator^31^ is used to model the optical stiffening of the RF tone and the dynamical shifts.
